# Focus on biomolecular condensates

**DOI:** 10.1093/plcell/koad182

**Published:** 2023-06-23

**Authors:** Emilio Gutierrez-Beltran, Lucia Strader, Peter V Bozhkov

**Affiliations:** Instituto de Bioquimica Vegetal y Fotosintesis, Consejo Superior de Investigaciones Cientificas (CSIC)-Universidad de Sevilla, Sevilla 41092, Spain; Departamento de Bioquimica Vegetal y Biologia Molecular, Facultad de Biologia, Universidad de Sevilla, Sevilla 41012, Spain; Guest Editor, The Plant Cell, American Society of Plant Biologists, USA; Department of Biology, Duke University, Durham, NC 27708, USA; Senior Editor, The Plant Cell, American Society of Plant Biologists, USA; Guest Editor, The Plant Cell, American Society of Plant Biologists, USA; Department of Molecular Sciences, Uppsala BioCenter, Swedish University of Agricultural Sciences and Linnean Center for Plant Biology, Uppsala 75007, Sweden

Biomolecular condensates, a term first coined by Banani et al. only 6 years ago (Banani et al. 2017), are nano- or microscale, intra- or extracellular assemblies that often form via liquid–liquid phase separation and have the ability to selectively concentrate or sequester biomolecules, primarily proteins and nucleic acids (Emenecker et al. 2021; Mitrea et al. 2022; Mountourakis et al. 2023). There is a remarkable compositional and structural diversity among biomolecular condensates we know to date, which range from classic membraneless organelles, such as nucleolus and pyrenoid, to single-component condensates made of the same type of protein molecules. This diversity of biomolecular condensates, together with their ubiquitous occurrence across all kingdoms of life and in response to a plethora of developmental and environmental signals, indicates that the condensation of proteins and nucleic acids was a key physicochemical process in the origin and evolution of biological traits. Immense interest in studying biomolecular condensates over the past several years has not bypassed plant biology, so releasing the first focus issue on this hot topic in *The Plant Cell* is timely.

The interaction between condensates and membranes is well-established in animal and yeast systems. However, our comprehension of the mechanisms by which condensates assemble and function on membranes is in its infancy, especially in plants. In a perspective article, Dragwidge and Van Damme (2023) focus on how membranes and membrane lipids regulate the formation and function of membrane-associated protein condensates and discuss relevant methodology.

Concentration or sequestration of proteins and/or nucleic acids in biomolecular condensates provides several elegant mechanisms for regulating the timing of developmental transitions as well as cell fate in response to hormonal and environmental cues. Field et al. (2023) review recent experimental evidence for the developmental roles of biomolecular condensates in the plant life cycle, discussing also the pros and cons of various techniques to study condensates, with particular relevance to plant developmental biology. Wang et al. (2023) provide new experimental evidence linking DECAPPING 5 (DCP5)—a component of processing bodies—to the control of flowering time. In this work, the authors found that condensation of DCP5 and its interacting partner SISTER of FCA is essential for transcriptional regulation of FLOWERING LOCUS C, one of the key factors responsible for the initiation of flowering in Arabidopsis.

Various stresses induce the formation of biomolecular condensates or engage constitutively present condensates, which help plants to adapt to and survive periods of stress. Solis-Miranda et al. (2023) provide a detailed overview of the assembly, molecular composition, physicochemical properties, mechanistic roles, and biotechnological applications of cytosolic stress-related condensates in plants, with a special emphasis on stress granules, processing bodies, and small interfering RNA (siRNA) bodies. Three original research articles report on aspects of stress-related condensates. Ruiz-Solani et al. (2023) report that targeting of a type I metacaspase to the proteotoxic stress-induced granules counteracts senescence by clearance of protein aggregates. Stress agents have been also shown to induce DNA damage, affecting plant growth and productivity. In this context, Yin et al. (2023) reveal that CROWDED NUCLEI 2, a lamin-like protein with multiple functions, undergoes phase separation in order to nucleate DNA repair machinery and promote DNA damage response. Finally, Hoffmann et al. (2023) report how viral protein machinery can modulate stress granules in the host plant cells. The authors demonstrate that *Cauliflower mosaic virus* protein P6 localizes to plant stress granules during infection and reduces their assembly.

RNA is found in several types of biomolecular condensates, and the interaction between RNA and RNA-binding proteins is known to promote phase separation. In this line of research, Kang and Xu (2023) review the emerging role of the N6-methyladenosine modification, the most widespread and abundant modification in eukaryotic mRNAs, in phase separation in plants.

Phase separation depends on a threshold of protein concentration, valency, and environmental parameters such as salt, pH, and temperature. This physical principle has been exploited by Safi et al. (2023) to generate a toolbox named SYnthetic Multivalency in PLants to study protein–protein interactions and kinase activities.

Besides the cytosol, biomolecular condensates are known to form inside membrane-bound organelles, being perhaps most abundant in the nucleus. Cajal bodies are one example of nuclear biomolecular condensates. Taliansky et al. (2023) first discuss evolutionarily conserved roles of Cajal bodies in the regulation of gene expression and telomere maintenance and then dwell on recent findings of unique plant-specific functions of Cajal bodies in posttranscriptional regulation under normal conditions and in responses to pathogen attack and abiotic stress.

Pyrenoids are a type of biomolecular condensate associated with a carbon-concentrating mechanism in the chloroplasts of most algae and hornworts, responsible for approximately one-third of global CO_2_ fixation. He et al. (2023) summarize the current understanding of pyrenoid function, structure, components, and dynamic regulation, including molecular mechanisms governing liquid–liquid phase separation of Rubisco together with essential pyrenoid component 1 (EPYC1) discovered in the model green alga *Chlamydomonas reinhardtii*. In their breakthrough report, Lau et al. (2023) have significantly advanced the methodological toolbox for deciphering molecular composition and suborganellar structure of pyrenoids and other phase-separated compartments in *Chlamydomonas* by demonstrating proximity labeling of pyrenoid proteins using TurboID.

Collectively, these articles showcase the breadth of both methodologies used to study biomolecular condensates and the fundamental implications of the condensates for plant biology. In this regard, biomolecular condensates are a dramatic example of a scientific area heavily reliant on a cross-disciplinary approach combining chemistry, physics, material science, cell, molecular and synthetic biology, and genetics, as well as machine learning and other branches of artificial intelligence and computer science. We anticipate that further progress in the understanding of molecular mechanisms regulating condensate assembly, biochemical reactions, and pathways occurring within biological condensates, and the physiological roles they play in plants will in the long run enable the engineering of biological condensates for enhanced crop fitness, resilience, and productivity. We encourage authors to continue to submit their best work on biomolecular condensates to *The Plant Cell*. Articles published in this area within 8 to 12 months of this focus issue will be added to an online collection on biomolecular condensates, building on the articles presented in this focus issue.
